# Psychosocial stress in families of young children after the pandemic: no time to rest

**DOI:** 10.1186/s13034-025-00905-5

**Published:** 2025-05-03

**Authors:** Katharina Richter, Catherine Buechel, Michaela Augustin, Anna Friedmann, Volker Mall, Ina Nehring

**Affiliations:** 1 Social Pediatrics, TUM School of Medicine & Health, Technical University of Munich, Heiglhofstr. 69, 81377 Munich, Germany; 2 German Center of Child and Adolescent Health (DZKJ), partner site Munich, Heiglhofstr. 69, 81377 Munich, Germany; 3 Kbo-Kinderzentrum, Heiglhofstr. 69, 81377 Munich, Germany

**Keywords:** Parent mental health, Child mental health, Infant regulatory problems, Emotional problems, Conduct problems, Parenting stress, Societal challenges, Crisis

## Abstract

**Background:**

During the pandemic, parenting stress and mental health challenges for both parents and children have increased. However, the lasting repercussions for families remain largely unexplored. Additionally, young families currently face stressors such as economic inflation, the Russia-Ukraine War, and the climate crisis, whose impacts on families are not yet understood. The primary aim of the study is therefore to evaluate parenting stress as well as child and parent mental health problems in the postpandemic era. Additionally, the study seeks to identify potential predictors of parenting stress and mental health issues in parents.

**Methods:**

From February 2023 to March 2024, we conducted a digital cross-sectional study involving families (*N* = 17,333) with children aged 0–9 years in Bavaria (Southern Germany) to examine parenting stress and mental health issues among both parents and children in light of current societal challenges. Validated questionnaires were used to gather data, and potential factors contributing to parenting stress were investigated.

**Results:**

We found that 53.7% of parents scored above the cut-off value, indicating that they experienced moderate to high levels of parenting stress. Additionally, 13.5% showed signs of anxiety symptoms, while 14.6% exhibited indications of depression according to cut-off values. Additionally, 34.9% of the infants (0–24 months) had crying and/or sleeping difficulties, whereas emotional and behavioral problems were observed in 8.7 of the toddlers (2–4 years) and 10.4% of the pre- and primary schoolers (˃ 4 years). Economic inflation was perceived as stressful or very stressful for 59.3% of parents, with radicalization and social division (49.3%), the Russia–Ukraine War (37.9%), and the climate crisis (31.8%) also cited as sources of stress. For 31.6% of families, the lingering effects of the pandemic continued to be a (major) burden. Across all age groups, children’s mental health issues and societal challenges were the primary predictors of parenting stress.

**Conclusion:**

Our study underscores that psychosocial stressors for families with children remain pronounced even postpandemic. Moreover, our findings highlight the impact of broader societal trends, such as economic inflation and social division, on family well-being. Addressing these stressors and promoting the mental health of infants while bolstering parental resilience by alleviating parenting stress should be key priorities for healthcare initiatives in the aftermath of COVID-19.

## Background

Parenthood usually brings happiness [1] but is challenging at the same time. Parents bear great responsibility for their child. They want to fulfil their children’s needs and often put their own on the back burner, which can result in parenting stress. Parenting stress refers to an imbalance between the demands of the parenting role and the available resources [2]. High parenting stress can lead to parental dysfunction [3] and can have several causes, such as high parental workload, low social support, the perception of the child as difficult, negative life events or problems with child caretaking. Higher maternal age (> 35), single parenting and having more than one child are also associated factors [4, 5]. In times of societal crisis, young families seem to react particularly vulnerable, as observed, for example, during the COVID-19 pandemic [6, 7]. Increased depression and anxiety rates have been reported in mothers and fathers during the pandemic [8–10], as has increased parenting stress [11–14]. Parenting stress is considered a decisive factor in the development of mental health problems in mothers and fathers [15–17], but it is also linked to impaired parent‒child interaction, especially with respect to limited parental emotional availability and a lower quality of caregiving in general [15, 18, 19]. Thus, parenting stress and parental mental health problems also affect children subsequently and are risk factors for their mental health [20, 21]. Compared with prepandemic data, significant increases in psychological problems in school children and adolescents during the COVID-19 pandemic have been reported [13, 22–25]. There is also evidence for an increase in crying, sleeping, and feeding problems in infants as well as highly pronounced behavioral and emotional problems in toddlers during the crisis [26].

Moreover, more than a year has passed since the WHO announced the official end of the pandemic in May 2023. However, the long course of the crisis and the accumulation of stress factors such as existential fears and worries, loss and grief, economic disadvantages, and negative personal and social changes in different life areas may still persist. These psychosocial stressors may leave lasting imprints and could result in serious health consequences [27–29]. Psychiatric research has consistently demonstrated that stressors may manifest as clinically significant disorders even years after exposure [30]. To date, there are few longitudinal data on the psychosocial situation of families in the postpandemic era. Even with the interim relaxations and the clear fading or complete termination of restriction measures in Germany in the course of 2022, the after-effects are likely to remain noticeable [31], raising the question of whether or how burden parameters are present in the meantime.

In addition to these potential lasting effects of the pandemic, further crises arose that might again specifically affect families in Germany. Recently, the existential threat posed by climate change, along with psychological distress and anxiety about potential future crises, has garnered significant attention in the literature. This research underscores that the perceived threat of climate change, beyond the immediate impact of natural disasters, constitutes a significant risk factor for mental health issues [27]. This might be more pronounced in young families since their future is more affected than the lives of the older generation.

While some studies have examined the immediate effects on those directly affected by the Russian–Ukraine War [32], few studies have addressed the mental health consequences of perceived threats in neighbouring countries, with greater anxiety in an adolescent population [33]. It stands to reason that, in addition to the immediate consequences of the war, the unpredictability of further developments might particularly affect young families in Germany due to the geographical proximity.

Economic inflation can have widespread negative effects on both society and individuals [34]. The established connection between socioeconomic conditions, stress exposure, and health demonstrates that these factors are closely linked. These socioeconomic stressors influence health through multiple mechanisms, especially via physiological and psychological stress responses [28]. In recent years, Germany has experienced a significant increase in living costs. It can be assumed that young parents are particularly affected by financial developments, as they are still building their livelihoods in addition to increased daily expenses.

While these challenges might not affect all families equally, a complex crisis constellation has arisen that has the potential to cause or intensify psychosocial stress [35–37]. Chronic stress, along with highly salient stressors typical of crises, can negatively impact mental health, leading to both short- and long-term adverse effects [38]. Symptoms of, e.g., anxiety and depression might be intensified by the omnipresence of these social crises in society and the media. This could already be observed in the context of climate change as well as political instability and war [39].

Despite existing evidence suggesting that the impacts of the climate crisis or financial inflation may serve as potential risk factors for mental health issues in adults or adolescents [28, 40], other extensive societal challenges like radicalization movements or social division, have not yet been taken into account. Often, direct influences of crises and disasters (e.g. in areas affected by climate disasters or military conflict zones) are examined as factors affecting psychological parameters, while more indirect effects are not explored. Furthermore, little is known about the influence of these current challenges on the experience of stress or mental health issues in parents of young children in particular. It still remains unclear how societal challenges interact with the stress experienced in the parental role and how this, in turn, affects mental health issues in parents. As part of the study project, the exploration of potential stress factors for parents of young children is broadened to include a social perspective.

Overall, we are currently experiencing the post-COVID-19 era coupled with other severe global and societal challenges, leading to the following research questions:

1. To what extent are parenting stress as well as parent and child mental health symptoms in young families pronounced in the postpandemic phase?
2. Which factors (sociodemographic, societal challenges and child mental health) are associated with parenting stress and parents’ mental health symptoms?

## Methods

### Study design

The JuFaBY (***Ju****nge****Fa****milien in****B****a****y****ern: Young Families in Bavaria*) study investigates psychosocial stress in families with young children (up to primary school age) in Bavaria (southern Germany). In the following, we present the results of a cross-sectional investigation. Data were collected from February 2023 to March 2024. The study protocol was approved by the Ethics Committee of the Technical University of Munich (vote no. 2022 − 483_1-S-KH).

## Participants

All participants were recruited and surveyed via the smartphone app “Meine pädiatrische Praxis” (“My pediatrician”) (www.monks-aerzte-im-netz.de), which is a well-established communication tool connecting parents with their pediatrician. For example, it sends out invitations for regular childhood check-ups. In Germany, preventive regular childhood check-ups (“U-Untersuchung”) take place at 3 weeks, 3 months, 6 months, 12 months and then annually until school age. Check-ups are recommended but not mandatory, and approximately 97% of parents make use of this offer [41]. To recruit participants for the JuFaBY study, an invitation was sent out via app coupled with the reminder for the next check-up at the respective age. All app users with children whose pediatric practices have not objected to a digital study invitation were invited to participate. Parents whose German language skills were not sufficient to understand the subject information were excluded from the study.

Study invitations, detailed information and informed consent were sent via app. In total, 345 pediatric practices agreed that their patients were invited, and approximately 152,000 invitations to parents were sent. Overall, 17,333 parents completed the study questionnaires.

## Measures

All the data were collected via standardized questionnaires via app. The participants were asked questions on their general sociodemographic characteristics, perceived societal stress factors, parenting stress and parent and child mental health outcomes.

## Sociodemographic characteristics

Caregivers were asked if they are mother or father of the child or any other caregiver (if yes, who). Furthermore, they were asked about their age, highest educational background, the monthly household income, the perceived financial status and whether mother and/or father of the child were born in Germany. Caregivers were further asked if they raise the child mainly alone (without partner), whether they have more than one child and how many hours per week the child is cared for in external care (“no parental care”, i.e. kindergarten, school etc.). They were asked if their child had a chronic condition or illness (yes/ no). Child’s birthday and sex are fix variables in the app. The answers were presented as corresponding alternative options, with an additional open-ended response choice.

## Perceived societal stress factors

Parents were asked a self-developed question about their perceived burden related to the following current societal topics (“How stressful are the following societal issues/events for you? “): climate change, Russia-Ukraine War, financial inflation, social division and radicalization, and the COVID-19 pandemic. The perceived burden was rated via 5-point Likert scales for each topic (from 1 = not at all stressful to 5 = very stressful).

### Parenting stress

To assess parenting stress, we applied the parent domain of the German adaptation of the ‘Parenting Stress Index (PSI)’ (“Eltern-Belastungs-Inventar” EBI) [42]. High scores indicate limited parental resources for upbringing and caring for the child. The parent domain includes the following subscales: ‘health’ (parental health impairment as a cause or a result of parenting stress), ‘isolation’ (lacking integration in social networks), ‘role restriction’ (perceived limitations as a result of being a parent), ‘parental competence’ (parental doubt about their own ability to manage upbringing and care for their child), ‘attachment’ (emotional relation of parent to the child), ‘depression’ (worries and negative thoughts due to the parental role) and ‘spouse-related stress’ (as a result of being a parent). Answers were given on a 5-point Likert scale ranging from 1 = strongly agree to 5 = strongly disagree, resulting in a possible score range of 28–140. The three cut-off categories for each subscale and the whole parent domain were ‘not stressed’ (T value < 60), ‘stressed’ (T value = 60–69), and ‘strongly stressed’ (T value ≥ 70). The internal consistency of the parent domain has been proven to be good (α = 0.93), and the retest reliability after one year has been shown to be r = 0.87. Correlations with stress indicators and related constructs have resulted in the assumption of test validity [43].

## Parental mental health

Current parental depression and anxiety symptoms were assessed with the very short screening tool Patient Health Questionnaire 4 (PHQ-4, German Version [44]). The questionnaire comprises two items each on the occurrence of depression and anxiety symptoms over the last two weeks. The four items are used to record the two diagnostic core criteria of major depression and generalized anxiety disorder. The response options on a 4-point Likert scale range from 0 “not at all” to 3 “almost every day”, resulting in a possible score range of 0–12. A cut-off of ≥ 2 for the depression items indicates depressive symptomatology, and a cut-off of ≥ 3 for the anxiety items refers to generalized anxiety symptomatology. The internal consistency of the PHQ-4 has been proven to be good (α = 0.85) [45]. The PHQ-4 showed acceptable reliability, with ω = 0.85. Construct validity was supported by intercorrelations with other self-reported scales and demographic variables [46].

## Infants’ crying, sleeping and feeding problems

For infants (0–24 months), the two subscales ‘crying/whining/sleeping’ and ‘feeding’ of the Questionnaire for Crying, Sleeping and Feeding (CSF; [47]) were applied. Parents answered 38 questions about the behavior of their infants. Answers were given on 4-point scales, and mean values were calculated (ranging from 1 to 4). According to validated cut-off values, the dichotomous outcomes ‘noticeable problems’ and ‘no problems’ were calculated for the domains ‘crying/whining/sleeping’ (cut-off value: 1.84, sensitivity: 87%, specificity: 92%) and ‘feeding’ (cut-off value: 1.27, sensitivity: 57%, specificity: 77%). The CSF also comprises questions to identify excessive crying as defined by the Wessel criterion (‘rule of three’) [48]. The reliability of the questionnaire has been confirmed by the high internal consistency of the scales [47].

### Emotional and behavioral problems of toddlers and pre- and primary schoolers

For toddlers (2–4 years) and pre- and primary schoolers (from 5 years), the Strengths and Difficulties Questionnaire (SDQ, short form of the German Version; [49]) was used to examine emotional and behavioral problems. Parents received the version that was appropriate for their child’s age (i.e., 2–4 years or older than 4 years). Parents were asked to classify the individual characteristics as not true, somewhat true or certainly true for their child in four domains (‘emotional symptoms’, ‘conduct problems’, ‘hyperactivity/inattention’, and ‘peer relationship problems’), resulting in a score ranging from 0 to 40 points. The cut-off values indicated that the child’s behavior was ‘no problems’ (0–13 points), ‘borderline’ (14–16 points) or ‘noticeable problems’ (17–40 points). The internal consistency has been shown to range between α = 0.73 and α = 0.86. By means of comparison with other corresponding scales (e.g., the Child Behavior Checklist), the validity of the instrument can be assumed to be acceptable [50, 51].

### Statistical analyses

For analyses, children were divided into three groups according to their U examination: “infants” (U4-U6, i.e., aged approximately 0–24 months), “toddlers” (U7-U8, i.e., aged approximately 2–4 years), and “pre- and primary schoolers” (≥ U9, i.e., aged 5 years and older).. Subjects with missing values were excluded from the analyses where necessary (see individual table descriptions). A descriptive analysis was performed on parenting stress (EBI), parent mental health (PHQ), as well as infant crying, sleeping, feeding (CSF), and behavioral and emotional (SDQ) difficulties. For the evaluation regarding societal challenges, the response options ‘not at all stressful’ and ‘not stressful’ were combined into ‘not (at all) stressful,’ and ‘very stressful’ and ‘stressful’ were combined into ‘(very) stressful’.

To include certain variables in the regression analyses, these variables were dichotomized as follows: education status was dichotomized into high (university degree and high school diploma) and low (secondary and lower secondary school diploma), and those cases whose response referred to ‘other qualification’ and thus could not be allocated to either of the groups were excluded from the analyses. Financial status, as measured by participants’ subjective perception that additional purchases are possible after basic needs are met, was also dichotomized into high (‘family income generally allows for very large additional purchases’ and ‘family income generally allows for large additional purchases’) and low (‘family income generally allows for only small additional purchases’, ‘family income generally allows for only very small additional purchases’, and ‘family income not sufficient to meet basic needs (no additional purchases possible)’. Participants who did not want to provide an answer regarding their current financial status were excluded from the analyses related to this outcome variable. Multiple linear regression models were constructed for the outcomes EBI total score T-value (i.e., parenting stress) and PHQ total score (i.e., parental mental health symptoms). Within the framework of the regression analysis, six models were developed to anticipate the two parental outcomes across all three distinct age groups (i.e., infants, toddlers, and pre- and primary schoolers). The following predictors were included: sociodemographic factors (education status, financial status, child age, having more than one child (i.e., having a sibling), being a single parent (i.e., raising the child mostly alone), child chronic illness and/or disability, external child care (“non parental care” in hours per week), perceived global psychosocial stressors (sum score of perceived burden of climate change, inflation, social division, Russia-Ukraine War and Covid-19 pandemic), CSF crying/whining/sleeping problem score, CSF feeding problem score, SDQ 2–4 years total score and SDQ age 5 + total score (i.e., child mental health problems). The models resulted in the calculation of standardized beta weights and their p values for corresponding predictor variables. Standardized beta weights (*β*) represent as measure of effect size with *β* ˂ 0.1 indicating a small, *β* ˂ 0.3 indicating a medium and *β* ˂ 0.5 indicating a large effect [52]. The requirements for calculating multiple linear regression models were met. All the described results were based on an alpha level of 5%. To control for multiple testing (n = 6), Bonferroni correction was performed. Analyses were performed in IBM SPSS Statistics Version 28.0 for Windows [53].

## Results

### Sample characteristics

The final cohort comprised 17,333 families. The average age of the parents was 35.2 years (*SD* = 5.3), with mothers averaging 35.0 years (*SD* = 5.2) and fathers 38.0 years (*SD* = 5.9) (Table 1).

**Table 1:** Characteristics of participants

Parents	%	*n*
Mothers	93.9	16,279
One or both parents born in Germany	95.9	16,616
Single Parent	9.3	1615
Level of education		
University degree	37.4	6475
High school diploma	18.0	3112
Secondary school diploma	32.0	5543
Lower secondary school diploma	10.1	1753
No diploma (yet)	0.8	131
Other qualifiations	1.8	307
Perceived Financial status		
Very large additional purchases possible	6.8	1183
Large additional purchases possible	35.7	6186
Small additional purchases possible	36.6	6337
Very small additional purchases possible	10.5	1820
No additional purchases possible	2.1	370
Not specified	8.2	1422
Monthly household income		
Less than 1500 €	2.8	489
1500–2499 €	9.6	1663
2500–3499 €	17.7	3076
3500–4499 €	21.3	3698
4500–5499 €	15.2	2626
5500 € or more	13.4	2327
Not specified	19.9	3454
External care		
Not at all	31.0	5376
Up to 10 h per week	17.3	2988
Up to 20 h per week	14.9	2580
Up to 30 h per week	21.9	3803
More than 30 h per week	14.8	2571
Children		
Infants	25.9	4489
Toddlers	40.8	7079
Pre- and primary schoolers	33.3	5765
Boys	51,4	8909
German mother tongue	94.6	16,397
Chronic illness and/or disability	5.8	1002
Siblings	57.7	10,000

*N* = 17,333

Table 1: Characteristics of the participants.

Notably, 93.9% of the participants were mothers. The infants were, on average, 5.0 (*SD* = 4.2; IQR 2.0–8.0) months old, the toddlers were 30.9 (*SD* = 10.1; IQR: 20–42) months old, and the pre- and primary schoolers were, on average, 82.0 (*SD* = 21.7; IQR: 59–101) months old.

Siblings were present in 57.7% of the families. More than half of the parents (55.4%) had attained at least a high school diploma, and 42.5% reported a perceived high financial status. In contrast, 47.1% of the families reported a low perceived financial status, and an additional 2.1% of the families indicated that their current financial status cannot cover the costs of basic necessities.

Most of the children (95.9%) had both parents or at least one parent born in Germany, and 99.4% of the children had resided in Germany since birth. Additionally, German was the predominant language within households, with 94.6% of the surveyed families indicating that German was spoken mainly at home.

### Perceived societal challenges

**Fig. 1:**
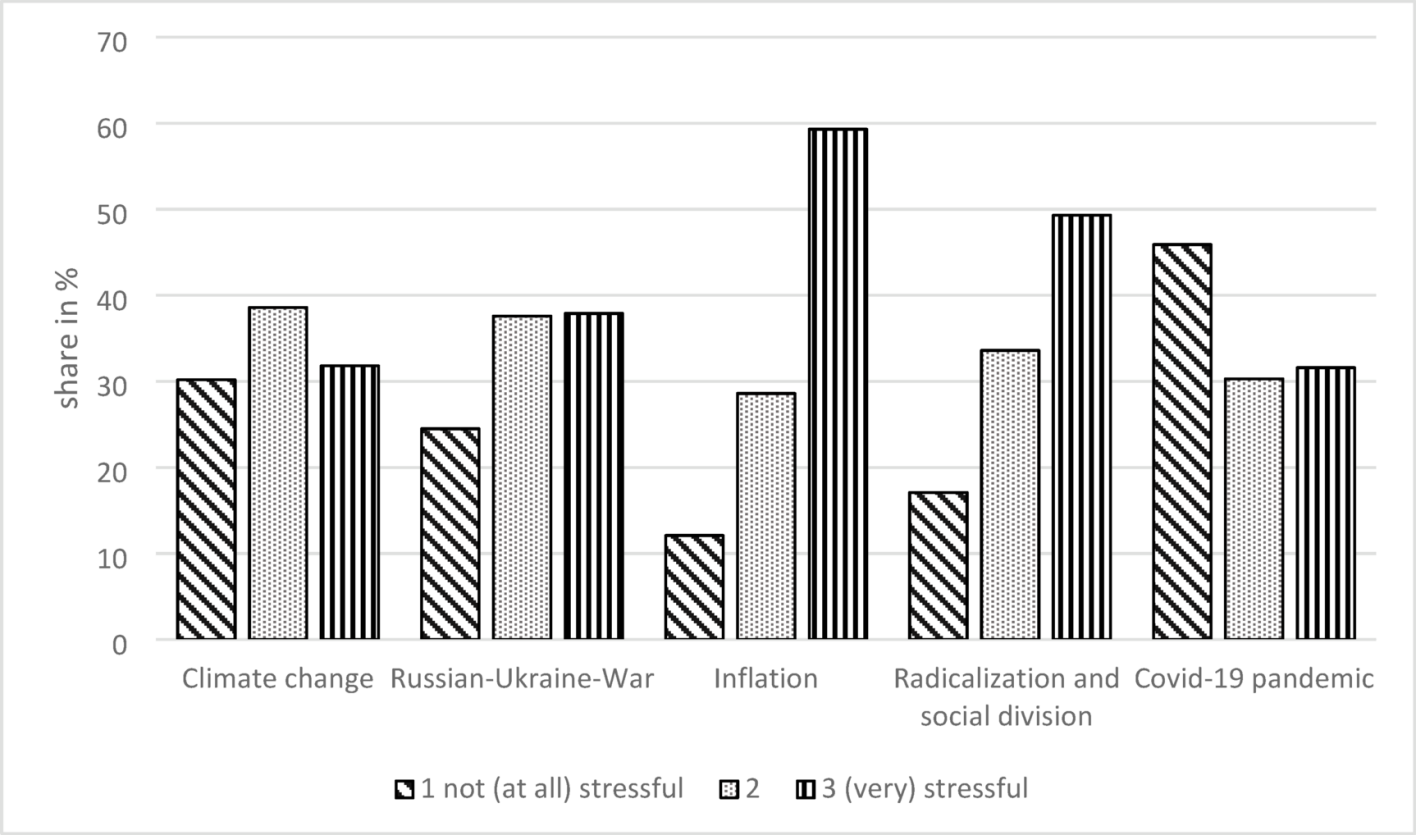
Perceived societal challenges

Financial inflation was perceived as “stressful” or “very stressful” by almost two-thirds (59.3%) of the respondents, whereas social division and radicalization were deemed (very) stressful by 49.3% of the parents (Fig. 1). Further challenges were perceived as (very) stressful by 37.9% (Russia-Ukraine War), 31.8% (climate change), and 31.6% (COVID-19 pandemic) of the parents.

### Parenting stress

According to the EBI (Table 2), 37.4% of the parents were ‘stressed’, and 16.3% were ‘strongly stressed’. Among the individual subscales of the EBI in the overall sample, ‘depression’ and ‘health’ stood out, with 68.7% and 58.9% of the participants scoring above the cut-off value for being ‘stressed’ or ‘strongly stressed’, respectively.

**Table 2:** Parenting stress and parent mental health

Parenting Stress Inventory (EBI)	%	N
Categorial evaluation of the parent domaina^a^		
No findings	46.3	7705
Stressed	37.4	6216
Strongly stressed	16.3	2713
Subscales (above cut-off)		
Attachment	33.8	5821
Isolation	53.3	9186
Parent competence	47	8099
Depression (worries and negative thoughts due to the parental role)	68.7	11,826
Health	58.9	10,142
Role restriction	47.7	8224
Spouse related stress^a^	47.8	7957
Parent mental health (PHQ)		
Total		
None	51.4	8851
Low	37.6	6477
Moderate	8.1	1397
Strong	2.9	499
Depression		
Inconspiciuous/normal	86.5	14,895
Noticeable/abnormal (probable)	13.5	2329
Anxiety		
Inconspiciuous/normal	85.4	14,706
Noticeable/abnormal	14.6	2517

^a^*N* = 16,634

### Parental mental health

Regarding their overall mental health, 48.6% of the parents reported concerns. Among them, 37.6% presented mild symptoms, 8.1% moderate symptoms, and 2.9% severe symptoms (Table 2). When the subscales were evaluated separately, 13.5% had symptoms of depression, and 14.6% had symptoms of anxiety. The overall mean PHQ-4 score was 2.82 (*SD*: 2.31).

### Child mental health

According to the CSF, 35.0% of the infants showed feeding problems, 34.9% had noticeable crying/whining/sleeping problems, and 6.1% met the Wessel criterion for excessive crying (Table 3). The overall SDQ for toddlers (pre- and primary schoolers) revealed that 9.8% (8.8%) of the children were in the ‘borderline’ range and that 8.7% (10.4%) were in the ‘noticeable’ range with respect to emotional and behavioral problems. In terms of the SDQ subscales, 15.2% (11.5%) of the parents reported peer problems, 10.0% (12.5%) reported hyperactivity, 12.2% (23.3%) reported emotional problems, and 26.0% (16.5%) reported problems for their children.

**Table 3:** Child mental health

Child mental health (CSF and SDQ)	%	*n*
Infants: Noticable crying, feeding & sleeping problems		
Excessive crying (Wessel criterion)	6.1	274
Crying/Whining/Sleeping	34,9	1529
Feeding	35.0	1536
*Pre- and primary schoolers: Emotional and behavioural problems (Categorial evaluation of SDQ total score)*		
Inconspiciuous/normal	80.8	4657
Borderline	8.8	508
Noticeable/abnormal	10.4	598
*Toddlers: Emotional and behavioural problems (Categorial evaluation of SDQ total score)*		
Inconspiciuous/normal	81.4	5764
Borderline	9.8	696
Noticeable/abnormal	8,7	617

*n*_*infants*_*= 4383*,* n*_*toddlers*_*= 7077*,* n*_*pre− and primary schoolers*_*= 5763*

### Predictive factors of parenting stress and mental health

Across all age groups, consistent and strong predictors of EBI emerged (Table 4). The strongest predictors were crying/whining/sleeping problems in infants (*ß* = 0.386, *p* < 0.001), emotional as well as behavioral problems in toddlers (*ß* = 0.361, *p* < 0.001) and pre- and primary schoolers (*ß* = 0.386, *p* < 0.001). Similarly, societal challenges consistently predicted higher parenting stress levels across all age groups (for parents of infants: *ß* = 0.169, *p* < 0.001; toddlers: *ß* = 0.174, *p* < 0.001; and pre- and primary schoolers: *ß* = 0.198, *p* < 0.001). External childcare was consistently associated with higher levels of parenting stress across different age ranges (for parents of infants: *ß* = 0.034, *p* = 0.012; toddlers: *ß* = 0.144, *p* < 0.001; and pre- and primary schoolers: *ß* = 0.147, *p* < 0.001).

**Table 4:** Linear regression analysis to predict the EBI total score T value and PHQ total score

Outcomes	EBI total score T value				PHQ total score			
	Infants							
	B	SE B	ß	*p*	B	SE B	ß	*p*
	*R*²= 0.270 *F* (11) = 150,214 *p* = < 0.001				*R*²= 0.205 *F* (11) = 104,998 *p* **= <** 0.001			
Parent Age	− 0.025	0.029	− 0.012	0.391	− 0.025	0.007	− 0.052	< 0.001*
Single Parent	0.801	0.487	0.021	0.100	0.419	0.114	0.050	< 0.001*
Siblings	2.124	0.284	0.103	< 0.001*	0.274	0.066	0.059	< 0.001*
External care	0.386	0.153	0.034	0.012	0.136	0.036	0.053	< 0.001*
Child chronic illness or impairment	− 0.275	0.901	− 0.004	0.760	0.403	0.211	0.026	0.056
Parent education (dichotomized)	− 0.3005E-5	0.001	0.000	0.973	0.000	0.000	0.007	0.587
Financial situation (dichotomized)	0.471	0.258	0.024	0.068	− 0.286	0.060	− 0.065	< 0.001*
Child Age	0.107	0.030	0.047	< 0.001*	0.015	0.007	0.029	0.038
Social challenges	0.476	0.037	0.169	< 0.001*	0.100	0.009	0.157	< 0.001*
Infant crying, whining, fussing	10.011	0.358	0.386	< 0.001*	1.605	0.0840	0.276	< 0.001*
Infant feeding	5.102	0.432	0.162	< 0.001*	1.257	0.101	0.178	< 0.001*
	Toddlers							
*R*²= 0.191 *F* (10) = 167,248 *p* **= <** 0.001					*R*²= 0.153 *F* (10) = 127,975 *p* **= <** 0.001			
Parent Age	0.043	0.022	0.022	0.051	− 0.009	0.006	− 0.018	0.123
Single Parent	0.237	0.355	0.007	0.504	0.615	0.094	0.073	< 0.001*
Siblings	0.343	0.203	0.019	0.092	0.101	0.054	0.021	0.061
External care	0.959	0.076	0.144	< 0.001*	0.114	0.020	0.066	< 0.001*
Child chronic illness or impairment	− 0.355	0.479	− 0.008	0.458	0.199	0.127	0.017	0.116
Parent education (dichotomized)	0.001	0.001	0.020	0.065	0.000	0.000	0.014	0.210
Financial situation (dichotomized)	0.158	0.203	0.009	0.437	− 0.342	0.054	− 0.072	< 0.001*
Child Age	− 0.047	0.010	− 0.053	< 0.001*	− 0.001	0.003	− 0.006	0.628
Societal challenges	0.463	0.029	0.174	< 0.001*	0.114	0.008	0.165	< 0.001*
Child emotional and behavioral problems	0.680	0.021	0.361	< 0.001*	0.143	0.005	0.295	< 0.001*
	Pre- and primary schoolers							
*R*²= 0.225 *F* (10) = 167,384 *p* **= <** 0.001					*R*²= 0.170 *F* (10) = 117,702 *p* **= <** 0.001			
Parent Age	0.062	0.024	0.032	0.011	− 0.003	0.006	− 0.006	0.666
Single Parent	0.761	0.364	0.025	0.037	0.656	0.091	0.090	< 0.001*
Siblings	1.109	0.290	0.045	< 0.001*	0.042	0.072	0.007	0.561
External care	1.055	0.092	0.147	< 0.001*	0.090	0.023	0.052	< 0.001*
Child chronic illness or impairment	− 0.830	0.385	− 0.026	0.031	0.327	0.096	0.042	< 0.001*
Parent education (dichotomized)	0.001	0.001	0.007	0.530	0.000	0.000	− 0.023	0.056
Financial situation (dichotomized)	1.078	0.243	0.054	< 0.001*	− 0.105	0.061	− 0.022	0.083
Child Age	− 0.021	0.006	− 0.048	< 0.001*	0.004	0.002	0.034	0.013
Societal challenges	0.564	0.034	0.198	< 0.001*	0.127	0.008	0.184	< 0.001*
Child emotional and behavioral problems	0.694	0.002	0.386	< 0.001*	0.128	0.006	0.294	< 0.001*

*p ≤ 0.0083 (Bonferroni adj. Alpha)

Significant predictors of a higher total PHQ score across all age groups were infant crying/whining/sleeping, respectively, and children’s emotional and behavioral problems. These factors had the highest effect sizes (*ß* = 0.28 − 0.29), followed by societal challenges (*ß* = 0.16- 0.20). Being a single parent (*ß* = 0.05 − 0.09) and using external care (*ß* = 0.05 − 0.07) were also significantly associated with the total PHQ score. Parental age (*ß* = 0.006 − 0.05) and financial status (*ß* = − 0.02 - − 0.07) were negatively associated with the total PHQ score.

## Discussion

This cross-sectional study of approximately 17,000 families revealed that more than half of the parents experienced moderate or high parenting stress, and 11% experienced moderate to strong depression and anxiety symptoms. One-third of the infants reported crying/whining/sleeping problems, more than one-third reported feeding problems, and some 10% of the older children reported emotional and behavioral problems. Child behavior and current societal challenges (e.g., economic inflation, the Russia-Ukraine War and climate change) were significantly associated with parenting stress and parental mental health symptoms.

The preceding CoronabaBY study examined a comparable population during the COVID-19 pandemic and reported a high prevalence of parenting stress compared with prepandemic data [54], which further increased from 38 to 51% during the pandemic, independent of restrictions [26]. This was confirmed by another pandemic study [55]. The present study revealed that more than 53% of the parents experienced parenting stress. In particular, the proportion of “strongly stressed” parents was significantly greater in the present survey (16%) than in the CoronabaBY study (11% during the pandemic) (data not shown). Additionally, as already observed in previous studies, most parents were affected by the subscales “health” and “depression”. High values on the “health” subdomain, as seen in 58% of the parents, indicate physical exhaustion and less energy. High values on the “depression” scale were present in more than two-thirds of the parents and indicated feelings of parental insecurity and guilt. This might lead to the risk for an impaired parent‒child relationship [42], as indicated in a third of the families (“attachment” subscale).

According to the PHQ subscales, 14.6% of the parents reported having anxiety symptoms, and 13.5% reported having depression symptoms. This figure is approximately 4% greater than that in the prepandemic general German population [56]. A German prepandemic study reported a mean PHQ-4 score of 1.58 for parents of children up to 4 years of age [57]. Another study reported a significant increase in the mean PHQ score from 2.3 to 2.5 in the first year of the pandemic in adults [58]. The present postpandemic study population had a mean value of 2.82. As initially expected, these findings indicate that pandemic-related stressors may have manifested and impacted long-term mental health in the population. Most likely, the current crises might additionally contribute to the constantly high parental strain.

Heightened parenting stress is associated with certain restrictions in parental functioning, which might have negative consequences for a healthy parent‒child relationship and sensitive parenting behavior [21]. Studies suggest that this, in turn, could facilitate the emergence or intensification of mental health problems in children [59–61]. In this study, more than one-third of the parents reported behavioral problems in their infants. Excessive crying was present in 6.1% of the infants, which is within the normal range [62, 63] and slightly greater than that reported in a previous study in which the same measurement tool was used [26].

Problems pertaining to sleeping and whining were observed in almost 35% of the infants, which is comparable to the findings of a previous study, where the proportion of these problems increased during the pandemic to 35.5% [26]. In this study, these higher rates are associated with higher rates of the abovementioned parenting stress and parental mental health problemsFeeding problems were present in approximately 35% of the infants, which is similar to pandemic data [26, 31].

Taken together, the rates of parenting stress, parental mental health symptoms and infants’ crying/whining and sleeping problems are still pronounced [26]. Constantly elevated stress levels due to the pandemic and subsequent ecological problems, the societal division added by the Russia–Ukraine war, climate change and global political troubles might amongst other factors contribute to hinder the relaxation of stress exposure.

In contrast, children aged 2 years and older did not have higher values of emotional and behavioral problems than did those in previous studies during the pandemic. The SDQ yielded 18.5% of the toddlers showing at least borderline behavior, which is in the normal range and almost at a prepandemic level (17–18%) [64]. Among pre- and primary schoolers, 19.2% reported behavioral conspicuities, which is less than during the pandemic [26]. One possible explanation is that the constantly inconspicuous rates of emotional and behavioral problems might be a result of children being older, being less dependent on their parents and potentially being less vulnerable to parenting stress than infants are. Additionally, it cannot be ruled out that the application of the SDQ, which aims to screen for psychiatric disorders more than observing smaller changes in child behavior, is the reason for the constant rates [49]. However, this should not be a reason to lose sight of these children, since current studies on children aged 11 years and older reported high levels of stress and worries due to the pandemic, climate change and the Russia-Ukraine-War [24, 33]. Therefore, preventive measures to avoid the occurrence of mental health problems in this generation should be initiated.

Although the population under study had average to high income [65], almost 60% perceived economic inflation as stressful or very stressful. A perceived good financial situation seems to be protective against mental health problems since it was negatively associated with parental mental health symptoms in parents of children up to 4 years of age. Surprisingly, this phenomenon occurred in parents of pre- and primary school-aged children, where a perceived good financial situation was positively associated with parenting stress. It is conceivable that a higher workload (i.e., more responsibilities in the job, compatibility of career and family) results in a greater burden and hence more parenting stress. Furthermore, radicalization and social division were among the most stressful current societal challenges (49.3%), followed by the Russian-Ukraine-War. These problems may touch families rather indirectly and are therefore not as challenging as direct-acting economic inflation. Yet, preliminary studies suggest that the more indirect effects of societal crises and challenges, such as fear of potential consequences from climate disasters and wars, can have a significant impact on mental health [28, 66].

As shown in the regression analyses, parenting stress and mental health symptoms had the strongest associations with child behavior problems with partly moderate effects across all age groups. There is evidence confirming the association between child psychological problems and parenting stress [21] since conspicuous child behavior may require more parental resources, leading to less energy and emotional availability, as indicated in the EBI subscales. However, child behavior problems are measured by parent reports and might hence be overestimated by burdened parents. It cannot be ruled out that, vice versa, parenting stress and mental health symptoms may impact a child’s behavior, as shown in previous studies [21].

Additionally, across all age groups, societal challenges were strongly associated with both parenting stress and parental depression and anxiety symptoms. The pervasive presence of these issues, particularly in the media, ensures that they inevitably impact families, even if they are not directly affected by these societal challenges. The widespread uncertainty, political debates, fears about the future, and growing divisions within society create an atmosphere of unease that extends into family life. Beyond this broader influence, families are also directly affected by immediate circumstances in their daily environment. For instance, rising living costs may force parents to work longer hours to meet financial demands, increasing the reliance on external childcare. This dynamic places families with lower incomes and limited financial security at particular risk, heightening parenting stress and the likelihood of mental health issues. Moreover, parents’ anxieties and concerns—whether related to political trends or societal divisions—may, in turn, shape their decision-making and interactions with their children. Yet, the observed effect sizes in this study are small and therefore their practical implication needs to be discussed with caution. Especially since to date, there has been weak evidence on the relationship between environmental factors and parenting stress or parent mental health issues. Thus, the results cannot be compared with other findings, which makes further research indispensable.

In parents of children aged 2 years and older, both parenting stress and mental health were significantly associated with external care. It is conceivable that parents are stressed because economic inflation forces them to work more hours, which in turn might force them to send their children to external care for a longer period of time. On the other hand, a recent study revealed that parenting stress was greater when external care was “formal”, i.e., not performed by family members (such as grandparents), and thus was independent from external rules and pressure [67]. This cannot be proven on the basis of our data. However, external care was highly correlated with parental working hours (data not shown), suggesting that more working hours may lead to increased parenting stress. In addition, the childcare situation itself could also lead to increased parenting stress. Against the backdrop of staff shortages, there has been an increase in absenteeism or reduced hours of care in childcare facilities across Bavaria. Due to the scarcity of childcare options, parents sometimes do not receive the desired placement or are forced to travel long distances. Altogether, this could lead to an increased perceived parenting burden, particularly for parents whose children spend extended periods of time in childcare facilities. Other factors, such as work-related stress, the balance between work and family, and feelings of guilt towards the child, may also explain the observed correlation. Relevant interrelations should be explored in more detail in future studies.

Being a single parent was significantly associated with parental mental health symptoms, which can be interpreted bidirectionally: mentally burdened parents might have more family conflicts, resulting in separation or divorce. In contrast, single parents have a greater mental load [68], which might cause symptoms of depression or even anxiety.

In summary, young families have experienced certain challenges and changes at different levels in recent years. According to Bronfenbrenner theory [69], such changes might influence a child’s development. For example, high parenting stress impacts a child’s microsystem. The macrosystem, i.e., culture, ideology, laws, etc., has changed due to the pandemic, the Russia–Ukraine War, and climate change. Hence, an impact of the surrounding systems on a child’s psychological development cannot be ruled out.

In light of the study results, it is clear that families currently face a significant need for support. This highlights the necessity of establishing easier access to support services for families. Child psychological difficulties are associated with increased parental stress and psychopathology, thereby impacting the overall functioning of the family system. Early counselling and therapeutic support are therefore absolutely essential. Additionally, burdened families require concrete relief in their daily lives, with childcare being of central importance in this regard. The next step should be to understand how childcare services are perceived as supportive, in order to, for example, ensure a better work-life balance. On a political level, there is also a need for action in terms of financial relief for families, which is currently perceived as burdensome by many. Societal challenges represent potential risk factors for the well-being of families and must be recognized as such. It is the responsibility of political actors to take this into account in their actions.

### Strengths and limitations

The study has a very large sample size, applied validated questionnaires and had very few missing data. This is the first study on the impact of multiple crises on families with infants and toddlers. With a sample size of *n* = 17,333, even very small effects could be found with a statistical power of 0.9 and a significance level of α = 0.05.

However, selection bias cannot be ruled out: the population comprised only app users and were almost exclusively German citizens living in Bavaria. Additionally, only a relatively small proportion of all parents invited to participate in the study actually took part in the survey, which may also have led to potential biases in the results. It is conceivable that only less burdened parents, who have the resources to participate in a study, would take part in the project. Furthermore the sample consisted primarily of respondent mothers. It can be assumed that this reflects the current care reality in families, as mothers continue to take on a large part of the care work [70]. According to the daily hassles theory, everyday challenges in dealing with the child in particular lead to increased parenting stress [71], which is therefore likely to be particularly pronounced among mothers. Additionally, the population had an education level similar to that of the average German population [72] but a rather high income, which limits generalizability and might lead to a slight underestimation of our results. Families with higher incomes, for example, could be less affected by the impact of inflation. It can also be assumed that families with a higher level of education have better resources, e.g. in the context of information and education in dealing with societal challenges, and can fall back on a better support network in the event of increased stress. In general, low-threshold survey options, e.g. in the form of apps, are particularly suitable for reaching as diverse a target group as possible in future. In addition to cooperation with pediatricians, collaboration with childcare facilities and schools could also be useful. In future, questionnaires should also be offered in different languages in order to be able to address families with different cultural backgrounds. All data collected in this study is based on information provided by parents, which may result in biased data as the parents’ level of burden might have an impact on the perception and reporting of the child’s symptomatology. Future projects should incorporate data from additional reference persons, such as a second primary caregiver. Moreover, the data should be enriched with insights from specialists, e.g. the child’s treating pediatrician. To explore the direction of certain effects, a cross-sectional survey is of course not ideal, as its explanatory power is limited and does not allow for causal inferences. In some cases, bidirectional interactions must also be considered. Within the context of family dynamics, it can be assumed that children’s psychological difficulties lead to increased parenting stress, which, in turn—potentially mediated by more dysfunctional parenting behaviors—could further reinforce the child’s symptoms. Parental anxiety and depressive symptoms are also potential risk factors for the development of psychological difficulties in children. Furthermore, it must be considered that parents experiencing heightened parenting stress or symptoms of anxiety and depression may perceive societal challenges as more distressing. It is also possible that moderating or mediating variables, such as personality traits, underlie both outcomes but could not be accounted for in this study. Not least, it remains unclear whether stressed parents are more likely to place their children in external childcare which could also explain the observed statistical correlation. Follow-up studies would not only allow for causal statements to be made but also enable the depiction of potential interactions, such as those between child psychological difficulties, parental psychopathology, and parenting stress. As such, they represent the essential research design to address these unresolved questions. The focus of the underlying analysis was primarily on the relationships between societal challenges and child psychological difficulties in the context of parenting stress and parental mental health issues. However, other child, parental, and family factors must also be considered, such as parental pre-existing conditions, child temperament, or family conflicts, which are highly relevant in this context and should be explored in future studies. Furthermore, the study leaves open the question of which resources and coping strategies families specifically have in dealing with societal challenges. This should be addressed in future studies. Qualitative data could be particularly valuable in gaining a deeper insight into the specific familial issues within the context of societal challenges, as well as relevant resilience factors.

## Conclusion

Families’ psychosocial stress rates were high during the pandemic and have not decreased ever since. Parenting stress and infants’ crying/whining/sleeping problems were still pronounced, whereas toddlers and children at preschool and primary school age did not show high rates of conspicuous behavior. Child mental health problems appear to be closely linked with parenting stress and parental mental health, highlighting the significance of the interplay within the family structure. As the families’ macrosystem (environment) is changing proximal family life seems to be affected directly. The significant associations between external childcare, parental working hours, and parenting stress should be a focal point of future analyses.

## Data Availability

The datasets generated and/or analysed during the current study are not publicly available but are available from the corresponding author upon reasonable request.

## Abbreviations

CSF: Questionnaire for crying, sleeping and feeding
EBI: Eltern-Belastungs-Inventar (German Version of the Parenting Stress Index (PSI))
SDQ: Strengths and difficulties questionnaire
PHQ 4: Patient health questionnaire 4
